# Effects of Supplementary Strength–Power Training on Neuromuscular Performance in Young Female Athletes

**DOI:** 10.3390/sports8080104

**Published:** 2020-07-24

**Authors:** Konstantina Karagianni, Olyvia Donti, Christos Katsikas, Gregory C. Bogdanis

**Affiliations:** School of Physical Education and Sports Science, National and Kapodistrian University of Athens, 17237 Athens, Greece; karagiannik97@gmail.com (K.K.); odonti@phed.uoa.gr (O.D.); ckatsikas@phed.uoa.gr (C.K.)

**Keywords:** female, adolescence, resistance training, plyometric training, strength training

## Abstract

This study examined the effects of a short-duration supplementary strength–power training program on neuromuscular performance and sport-specific skills in adolescent athletes. Twenty-three female “Gymnastics for All” athletes, aged 13 ± 2 years, were divided into a training group (TG, *n* = 12) and a control group (CG, *n* = 11). Both groups underwent a test battery before and after 10 weeks of intervention. TG completed, in addition to gymnastics training, a supplementary 7–9 min program that included two rounds of strength and power exercises for arms, torso, and legs, executed in a circuit fashion with 1 min rest between rounds, three times per week. Initially, six exercises were performed (15 s work–15 s rest), while the number of exercises was decreased to four and the duration of each exercise was increased to 30 s (30 s rest) after the fifth week. TG improved countermovement jump performance with one leg (11.5% ± 10.4%, *p* = 0.002) and two legs (8.2% ± 8.8%, *p* = 0.004), drop jump performance (14.4% ± 12.6%, *p* = 0.038), single-leg jumping agility (13.6% ± 5.2%, *p* = 0.001), and sport-specific performance (8.8% ± 7.4%, *p* = 0.004), but not 10 m sprint performance (2.4% ± 6.6%, *p* = 0.709). No change was observed in the CG (*p* = 0.41 to 0.97). The results of this study indicated that this supplementary strength–power program performed for 7–9 min improves neuromuscular and sport-specific performance after 10 weeks of training.

## 1. Introduction

Long-term athlete development models provide general frameworks to prepare youth for sports and a physically active lifestyle [1]. These models aim to align practice with growth, maturation, and early sport specialization and to consider factors such as injury risk [2,3] and the limitations of the existing training practice schedules [3,4]. Muscular strength and power, speed, and agility are central fitness components in all long-term athlete development models, which propose participation in high-intensity physical activity and muscle strengthening exercises at least 3 times a week [3,5]. Muscular strength and power increase with age in boys and girls until the onset of puberty [6,7], while after this age, a plateau in muscular strength is typically observed in girls [8]. Sprint speed increases in early childhood (5–9 years) and shows accelerated improvement in boys ~12 to 14 years; however, improvements occur earlier in girls, and sprint performance reaches a plateau 2 to 3 years earlier than boys [9,10].

Resistance and plyometric training are efficient methods to improve strength, power, speed, and general athleticism in youth athletes [11,12]. Importantly, enhanced physical fitness is a prerequisite for motor competence and technical skill acquisition, in youth athletes [1]. Recently, Lesinski et al. [13] demonstrated the positive effects of resistance training (including weight-bearing exercises) on muscular strength and jumping performance in youth athletes, and especially in adolescents, with boys improving more than girls [13,14]. Plyometric training is also an effective method to increase lower-limb strength and power, sprinting, and change of direction abilities in young male athletes [15,16]. However, research is lacking in female athletes and in individual sports [17]. In a recent meta-analysis examining the effect of plyometric training in female athletes (8–18 years), Moran et al. [18] reported small-to-moderate effects on jumping performance, with larger effect sizes observed in younger (<15 years; ES = 0.78) compared to older athletes (>15 years; ES = 0.31). Nevertheless, limited research examined the extent to which young female athletes increase strength and power following different modes of training. This would be especially useful in sports such as gymnastics, where girls train and compete from a very early age [19], and increased physical fitness is associated not only with sports performance, but also with reduced injury occurrence.

“Gymnastics for All” is an early specialization sport, which incorporates various elements (artistic, rhythmic, acrobatic, and aerobic) executed by a group of athletes on the gymnastics floor. Performance of these complex skills requires high levels of relative strength, power, and flexibility [19,20,21,22], while aerobic capacity has also been found important for both competition scores and recovery [23,24,25] throughout training and competition. Due to the less competitive character of “Gymnastics for All” (group contest or festival), athletes typically train 3–4 times per week for 1.5–2 h per session. Gymnastics coaches often use combinations of skills repetitions in a circuit type of training, to improve athletes’ neuromuscular fitness [26,27]. However, while this training is commonly used in gymnastics to develop sport-specific fitness, the addition of an age appropriate, supplementary, strength and power training program may offer positive outcomes that surpass the benefits obtained by skills training alone [26,28]. Previous studies suggested that supplementary strength and power training using basic movement skills, may enhance technical competency, correct movement patterns, and reduce injury risk [26,29] especially in adolescent female athletes, who show decreased strength and increased injury risk compared to males [30,31]. An important limitation of the existing training practice schedules in most youth sports is that training time per session (typically 1.5 h) may not be sufficient for learning new skills and improving muscular fitness. However, there is limited information on possible modifications needed in such training schedules in order to conform to the guidelines of current long-term athlete development models [1]. For example, Moeskops et al. [26] used two weekly neuromuscular training sessions that lasted 35 min each, during an 8 week period and found increased leg stiffness and muscular endurance in 8–9-year-old gymnasts. However, such durations of fitness training may not be feasible in most youth sport training schedules, due to time restrictions. Thus, it may be useful to examine shorter-duration fitness interventions.

Recently, it was suggested that a combination of two or more types of training may be more efficient to improve fitness and sport performance of youth athletes [32,33,34]. For example, a combined resistance and plyometric training program, performed for 6 weeks enhanced maximal strength, countermovement jump height, and sprint speed in three groups of youth basketball players (13–15, 15–17, and >17 years), although the training program used in that study was less effective as the age of the basketball players increased [32]. However, the effects of such combined strength and power programs in younger athletes are still unclear. Thus, the aim of this study was to examine the effects of a 10 week, short-duration, supplementary strength–power training program on neuromuscular performance and sport-specific skills in female, adolescent “Gymnastics for All” athletes. It was hypothesized that this supplementary strength–power training would improve physical fitness and in turn performance in sport-specific skills, more than gymnastics training alone.

## 2. Materials and Methods

### 2.1. Participants

Power analysis indicated that a minimum of 6 gymnasts should be included in the study in order to detect an effect size (ES) of 0.78, obtained from the meta-analysis of Moran et al. [18] for girls younger than 15 years of age (within–between analysis of variance power = 0.80, alpha = 0.05, correlation between repeated measures *r* = 0.5; G-Power 3.1.9.2).

Twenty-six female “Gymnastics for All” gymnasts, aged 13 ± 2 years were recruited from one gymnastics club. Criteria for inclusion were: regular training (i.e., three times per week for 90 min per session) for at least two years under the same coach; no involvement in any systematic strength and power training; competitive experience of at least one year. Participants were excluded if they had any musculoskeletal injury in the last six months or if they missed >10% of the training sessions. Gymnasts were randomly allocated (allocation ratio 1:1) to a training group (TG) and a control group (CG). Three athletes (two from the training group and one from the control group) were excluded from the study because they did not complete all tests. The final number of participants along with their anthropometric and maturity characteristics are presented in Table 1. The maturity offset was estimated according to the prediction equation of Mirwald et al. [35]. Before the start of the study, researchers informed coaches, athletes, and their parents about the purpose and risks of the study, and an informed consent was signed by the athletes and the participants’ parents. All procedures were approved by the local university ethics committee (reference number: 115/10-04-2019) in compliance with the Code of Ethics of the World Medical Association (Helsinki declaration of 1964, as revised in 2013).

### 2.2. Study Design

A repeated-measures parallel group design was used in the present study. The same battery of tests was evaluated in both groups at the beginning and at the end of the intervention (10 weeks). Athletes in the TG performed a circuit-type strength and power training program (duration: 7–9 min) for 10 weeks in addition to their regular gymnastics training. The study took place in the pre-season period (from October to December). The following tests were repeated at baseline and after 10 weeks of training: 10 m linear sprint speed, one-leg (one-leg CMJ) and two-legs countermovement jump (CMJ), drop jump (DJ), single-leg jumping agility test (JA), and 10 consecutive repetitions of a sport-specific skill (round-off). All measurements were performed in the same testing session, 48 h after the last training. A standardized warm-up preceded testing that included 6 min of light jogging, dynamic stretching for the major muscle groups, and 2 short accelerations.

Twenty-six sessions of this circuit strength and power training program were performed on non-consecutive days (Monday, Wednesday, and Friday), at the end of regular gymnastics training. Four familiarization sessions were performed over a two-week period, before baseline testing, to get participants habituated to the circuit strength-power training and the testing procedures. Both training and control groups participated in all familiarization sessions. During that period, data were collected to calculate intraclass correlation coefficients (ICCs) for each test, using a two-way mixed-model analysis of variance (ANOVA).

### 2.3. Methodology

#### 2.3.1. Anthropometry

Body mass (to within 0.1 kg, Seca 700, Seca Ltd., Birmingham, UK), standing height, and sitting height (to within 0.1 cm, Charder HM-200P, Charder Electronic Co., Ltd., Taichung City 412, Taiwan) were measured for both groups.

#### 2.3.2. Vertical Jump Height

Vertical jump height was determined from flight time using an Optojump system (Microgate, SRL, Bolzano, Italy) [36]. Participants were instructed to jump at maximal effort with their hands akimbo, take off with the ankles and knees fully extended and land balanced on the same spot. During all jumping tests participants wore gymnastics shoes. For the one- and the two-leg CMJ, gymnasts were instructed to perform a countermovement (knee angle approx. 90°) and then immediately jump up. Trials were separated by 30 s and there was a 2 min rest between the different jump tests. For the single-leg CMJ test, the sum of the right- and left-leg jumps was calculated and used for further analysis. ICC for the two-leg CMJ was 0.93 (95% confidence interval (CI): 0.86–0.97) (standard error of measurement (SEM) = 3.3%, meaningful detectable change at 90% confidence interval (MDC_90_) = 2.17 cm) and for the sum of the right- and left-leg CMJ it was 0.94 (95% CI: 0.86–0.97) (SEM = 3.6%, MDC_90_ = 2.49 cm).

For the DJ, gymnasts stepped horizontally off a 20 cm box on the gymnastics carpet and then immediately performed a maximal rebound vertical jump with minimal ground contact time. The ICC for the DJ height was 0.98 (95% CI: 0.96–0.99) (SEM = 1.6%, MDC_90_ = 1.00 cm).

#### 2.3.3. Single-Leg Jumping Agility Test

As for the single-leg jumping agility, a cross hop was used. This test examines jumping agility, as it requires the participants to hop as fast as possible and to move in multiple directions [37]. Subjects had to perform five consecutive rounds of hopping for each leg as fast as they could. For this test, a cross consisting of five equal-sized squares (30 cm side) was marked on the gymnastics floor with tape. The distance of the front, back, right, and left square from the middle (central) square, was set at 20% of the participant’s body height, measured from the center of the middle square, to the center of all the other squares. The starting point of the test was the central square, where gymnast stood on one leg, and from there they had to jump forward, backward, to the right. and to the left, into the respective squares, always returning to the central square after each jump (one round). The total time to complete the 5 rounds was recorded electronically. When a gymnast touched the contralateral foot on the ground, or hopped in the wrong direction, they had to repeat the trial. Two trials interspersed by 5 min of rest were performed, and the best trial was used for further analysis. The average value of the right- and left-leg performance was calculated for further analysis. The ICC for single-leg jumping agility was 0.97 (95% CI: 0.93–0.98) (SEM = 1%, MDC_90_ = 0.62 s).

#### 2.3.4. Sprint Test

Ten meter linear sprint performance was assessed electronically (Microgate, SARL, Bolzano, Italy) [36]. Participants were asked to stand in an upright stride stance with the preferred leg forward, 0.3 m before the first infrared photoelectric gate, which was placed 0.75 m above the ground to ensure it captured trunk movement and avoided false limb motion signals. Two trials interspersed by 5 min of rest were performed, and the best trial was used for further analysis. The intraclass correlation coefficient for the 10 m sprint was 0.99 (95% CI: 0.98–0.99) (SEM = 0.3%, MDC_90_ = 0.015 s).

#### 2.3.5. Sport-Specific Skill

Round-off is a basic movement in gymnastics used to gain speed before performing a series of flic-flacs and saltos [38]. The round-off includes the following phases: (a) a run-up phase, which ends by placing the hands on the floor in a T-shape, while inverting the body; (b) the main phase of support, followed by a rapid push-off from the hands and snap down; and (c) the last phase in which the feet land together on the floor while the body is inverted [38]. Participants performed 10 consecutive repetitions of round-off as fast as possible, each one starting from two steps. The total time to complete the 10 repetitions was measured electronically. Two trials interspersed by 5 min of rest were performed, and the best trial was used for further analysis. The intraclass correlation coefficient for the 10 round-offs was 0.99 (95% CI: 0.98–0.99) (SEM = 0.7%, MDC_90_ = 0.42 s).

#### 2.3.6. Strength and Power Training

At the end of the training session (Monday, Wednesday, and Friday), gymnasts of the TG performed a circuit strength and power training program while at the same time the athletes of the CG performed body posture movements. This circuit strength and power program included two 5 week training blocks. Six strength and power exercises of progressive difficulty for arms, torso, and legs were performed in the first training block (15 s work–15 s rest), while the number of exercises was decreased to four and the duration of each exercise was increased to 30 s (30 s rest) after the fifth week (Table 2). Each training included a combination of strength and power exercises targeting major muscle groups (Table 2). The exercises included are presented in Table S1. Athletes were instructed to perform as many repetitions as possible during the time available for each exercise. Strength and power exercises were performed on the surface of a gymnastics carpet with the gymnasts wearing gymnastics shoes. Training was supervised by an experienced coach, and proper technique of movement was emphasized at every training and testing session.

### 2.4. Statistical Analyses

Descriptive statistics were calculated for all performance and anthropometric tests. The normality of data distribution and homogeneity of variance were checked using Shapiro–Wilk test and Levene’s test, respectively. Unpaired t-tests were applied to determine significant differences in baseline values between groups. A two-way analysis of variance (AΝOVA) [group (training/control) × time (pre/post-training)], with repeated measures on time, was conducted to examine the effect of strength and power training on all the examined parameters. When a significant main effect or interaction was observed (*p* < 0.05), a Tukey’s post hoc test was performed. Effect sizes (ES) for the ANOVA were determined by partial eta squared (η^2^). Partial eta squared (η^2^) values were classified as small (0.01 to 0.059), moderate (0.06 to 0.137), and large (>0.137). For pairwise comparisons, ES was determined by Cohen’s *d* [39] (small: >0.2, medium: >0.5, and large: >0.80). The intraclass correlation coefficient (ICC) was calculated using a two-way mixed model, to measure reliability for all measures. Additionally, the standard error of measurement (SEM) and the meaningful detectable change at 90% confidence interval (MDC_90_) were calculated. Statistical significance was set at *p* < 0.05. All statistical analyses were conducted using SPSS (IBM SPSS Statistics, Version 22.0, IBM Corporation, Armonk, New York, USA).

## 3. Results

### Performance Parameters

No statistical differences were observed between groups in baseline values (Table 1). Group × time interactions were found for all the examined parameters (*p* < 0.038) except for 10 m sprint speed (*p* = 0.709) (Table 3). Comparison of changes in performance values between the TG and CG showed large effect sizes, indicating improvement in one- and two-legs countermovement jumps, drop jump, single-leg jumping agility, and round-off performance time in the TG (Table 3).

In particular, significant group × time interactions were observed for two- and one-leg countermovement jumps height (*p* = 0.004, η^2^ = 0.329 and *p* = 0.002, η^2^ = 0.351, respectively) with the post hoc showing an improvement only for the TG (*p* = 0.032, *d* = 1.20 and *p* = 0.003, *d* = 0.73, respectively) (Table 3). Significant group × time interactions were also observed in round-off performance time and single-leg jumping agility (*p* = 0.004, η^2^ = 0.328 and *p* = 0.001, η^2^ = 0.398, respectively) with the post hoc test showing improvement only for the TG (*p* = 0.0109, *d* = 1.20 and *p* = 0.001, *d* = 2.15). A significant group × time interaction was also observed for drop-jump (*p* = 0.038, η^2^ = 0.189) with the post hoc test showing improvement only or the TG (*p* = 0.008, *d* = 0.98).

**Figure 1:**
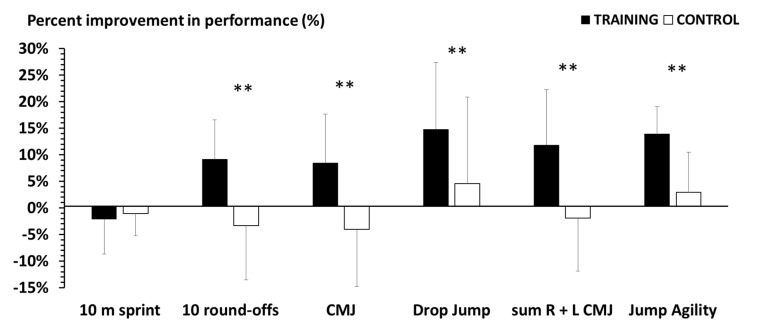
Percentage of change pre−post intervention in the examined parameters in the training and the control groups; CMJ: counter movement jump; R + L CMJ: sum of right– and the left−leg counter movement jumps; DJ: drop jump; ** *p* < 0.01 between training and control groups.

The percent changes in performance for the TG and the CG are presented in Figure 1. TG improved CMJ performance with one-leg (11.5% ± 10.4%, *p* = 0.002) and two-legs (8.2% ± 8.8%, *p* = 0.004), DJ performance (14.4% ± 12.6%, *p* = 0.038), single-leg jumping agility (13.6% ± 5.2%, *p* = 0.001), and sport-specific performance (8.8% ± 7.4%, *p* = 0.004), but not 10 m sprint performance (2.4% ± 6.6%, *p* = 0.709). All performance changes for the CG were not significant (*p* = 0.41 to 0.98).

## 4. Discussion

The main finding of this study was that a 10 week circuit-type strength and power training program of short duration (7–9 min) was effective in increasing jumping height, single-leg jumping agility, and sport-specific skill performance time in adolescent female gymnasts. Ten meter sprint speed remained unchanged in both groups, suggesting that this training program, as well as gymnastics training alone, did not improve speed abilities in adolescent female gymnasts. To the authors’ knowledge, this is the first study to examine a short-duration combined strength and power training program on jumping abilities, sprinting, and sport-specific parameters in young adolescent gymnasts. Importantly, the participants of this study were younger than 15 years, which is the age at which full adult height is typically achieved in females, thus representing a population undergoing maturational change [40].

The fact that a very short-duration (7–9 min) circuit-type program aiming to enhance whole-body strength and power training was so effective in improving jumping abilities, single-leg jumping agility, and sport-specific skill performance time in adolescent gymnasts, is of high practical value. This is because in most youth sports, athletes train 2–4 times per week for a limited time on each session (usually 1.5 h). During this restricted training time, coaches should allocate the necessary time to improve technical skills, team tactics, and physical fitness. Although previous research has shown that plyometric or strength and power training programs of longer duration (25 to 45 min) enhanced lower-limb strength and power in competitive gymnasts [26,41,42], such fitness training duration may not be feasible in most popular child sports, where technical and tactical training is prioritized. Taking this into account, a short-duration, whole-body strength and power program, such as the one used in the present study, would offer an efficient way to improve strength, power, and sport-specific skills, with minimal time investment.

The magnitude of improvements in single- and double-leg CMJ and in the DJ (Table 3, Figure 1), with effect sizes between 0.52 and 0.98, are in line with the findings of Moran et al. [18], who reported that vertical jump ability is developed to a greater degree in younger (<15 years) than in older (>15 years) female athletes (*d* = 0.78 and *d* = 0.31, respectively). When comparing the improvement of vertical jump between the TG and the CG in the present study, the effect sizes were large (1.01 to 1.47), indicating the effectiveness of this short-duration training program. Notably, this program improved not only general but also sport-specific fitness. In contrast, typical gymnastics training of the same weekly frequency and duration (3 times per week for 1.5 h per session—control group) does not seem to be an adequate stimulus for lower-limb power and sport-specific agility in adolescent athletes. This finding of the present study is important because it demonstrates the beneficial effects of a short-duration, supplementary, strength and power exercise program in these young athletes.

Single-leg jumping agility in this study was measured with a test that requires fast hopping forward, backward, and in the sagittal plane [37]. Single-limb hopping tests are typically testing functional ankle instability and are also classified as agility maneuvers due to the sudden direction changes that are required [37]. The ankle joint is often injured in gymnastics due to rapid take-offs and landings from different heights [43]. Several previous studies examined the role of strength and power training as a means of injury prevention in female athletes [44,45,46], but only a limited number of studies examined this relationship in female youth. Moran et al. [18] argued that a higher level of physical fitness not only increases performance but may also offset injury risk in child athletes. The strength and power program implemented in this study included exercises for arms, legs, and torso strength and power, thus, enhancing balance, coordination, and speed which would, in turn, improve single-leg jumping agility [34]. The pattern of large between-group differences was also evident in this test (between groups, *d* = 1.70) showing that sport-specific-skills repetition alone is not an adequate stimulus to improve a functional test associated with injury variables.

A running speed of at least 6.20 m·s^−^^1^, attained during 20 m run-up, is required for effective execution of a stretched salto [26,47]. Thus, the ability to accelerate, as well as the ability to execute fast technical “transition skills”, such as the round-off, is decisive for successful and safe performance of acrobatic elements in gymnastics. In the present study, athletes in the TG improved the time of execution of the round-off, possibly due to improved strength and power of the upper and lower limbs, as well as of the torso [42]. This would suggest that this brief strength and power training induces adaptations that are transferred to sport-specific skills, and thus it may be recommended for young female athletes of this sport. However, the ability to accelerate, as reflected by the 10 m sprint performance, remained unchanged in both TG and CG (Table 3). This finding is in contrast with previous research in younger female gymnasts (8–9 years old), which showed a significant but moderate improvement in 10 m performance (*d* = 0.40) after 8 weeks of plyometric training [41]. One possible explanation for this discrepancy may be a difference in trainability between these two age groups of athletes. In the present study, female athletes’ age was between 11 and 15 years, while in that previous study [41] female gymnasts were 8–10 years old. In child female athletes, the age before growth spurt (i.e., before the age of 11 years) is known as a “window of opportunity” for speed training [4,9], while a plateau in the trainability of sprint speed is observed in females at the ages of 11–14 [9,10], along with a concomitant increase in height and body mass. Thus, it is possible that an improvement in sprint performance may be more difficult to attain in adolescents between 11 and 15 years of age. Alternatively, the lack of improvement of 10 m sprint time may suggest that this low training volume is not enough to improve sprint performance over short distances, which require repetitive powerful muscle actions [48]. One limitation of this study is that performance in longer sprint distances was not examined. Therefore, it cannot be excluded that longer sprint performance (e.g., 20 m) would have been improved, as previous studies have found a twofold greater increase in 20 m compared to 10 m sprint performance following plyometric training (*d* = 0.40 vs. 0.81, respectively) [41]. Another limitation of this study was that in the sport-specific skill, only the time of execution was analysed. Further research should also analyse technical parameters of sport-specific skills to examine the transfer of improved strength and power on technique. In addition, future research should consider longer training interventions and in different periods over the year to examine strength and power adaptations in adolescent athletes, including males who may have different responses compared with female athletes at this age.

## 5. Conclusions

In conclusion, a brief-duration, supplementary, circuit-type strength and power training program was effective in increasing single- and double-leg vertical jump, jumping agility, and sport-specific skill performance in adolescent female gymnasts. Improvement of performance by devoting only 7–9 min per session may be an attractive model of supplementary training in female adolescent athletes, which may also reduce injury risk. The fact that gymnastics skills training alone was not adequate to improve important fitness and sport-specific parameters, may indicate that a supplementary strength and power training program is necessary for an effective and safe athlete development in this age group. Practitioners should consider incorporating a short-duration, supplementary strength–power program in “Gymnastics for All” training, as it was shown that substantial gains in neuromuscular performance can be obtained with minimum time investment.

## Figures and Tables

**Table 1:** Characteristics of the participants in the training group (TG) and the control group (CG) (mean ± SD).

Characteristics	TG (*n* = 12)	CG (*n* = 11)	*p*
Age (year)	13.2 ± 1.3	12.3 ± 1.3	0.106
Training experience (year)	4.4 ± 2.7	4.3 ± 2.1	0.883
Height (cm)	157.3 ± 6.1	156.0 ± 7.3	0.638
Body mass (kg)	52.4 ± 6.6	51.6 ± 8.5	0.792
BMI (kg/m^2^)	21.1 ± 1.6	21.2 ± 3.0	0.956
Maturity offset	1.1 ± 0.9	0.6 ± 0.9	0.159

**Table 2:** Strength and power training program executed three times per week for 10 weeks by the athletes of the training group (TG). Athletes performed two rounds of six exercises in a circuit form, for the first 6 weeks and two rounds of four exercises for the last 4 weeks.

	MONDAY	WEDNESDAY	FRIDAY
**WEEK 1**	2 × 6 exercises (5 S + 1 P)	2 × 6 exercises (5 S + 1 P)	2 × 6 exercises (5 S + 1 P)
	W:R = 15:15	W:R = 15:15	W:R = 15:15
	total duration: 7 min	total duration: 7 min	total duration: 7 min
**WEEK 2**	2 × 6 exercises (2 S + 4 P)		2 × 6 exercises (4 S + 2 P)
	W:R = 15:15		W:R = 15:15
	total duration: 7 min		total duration: 7 min
**WEEK 3**	2 × 6 exercises (1 S + 5 P)	2 × 6 exercises (3 S + 3 P)	2 × 6 exercises (1 S + 5 P)
	W:R = 15:15	W:R = 15:15	W:R = 15:15
	total duration: 7 min	total duration: 7 min	total duration: 7 min
**WEEK 4**	2 × 6 exercises (1 S + 5 P)	2 × 6 exercises (4 S + 2 P)	2 × 6 exercises (4 S + 2 P)
	W:R = 15:15	W:R = 15:15	W:R = 15:15
	total duration: 7 min	total duration: 7 min	total duration: 7 min
**WEEK 5**	2 × 6 exercises (6 P)		2 × 6 exercises (1 S + 5 P)
	W:R = 15:15		W:R = 15:15
	total duration: 7 min		total duration: 7 min
**WEEK 6**	2 × 6 exercises (1 S + 5 P)	2 × 6 exercises (2 S + 4 P)	2 × 6 exercises (1 S + 5 P)
	W:R = 20:20	W:R = 20:20	W:R = 20:20
	total duration: 9 min	total duration: 9 min	total duration: 9 min
**WEEK 7**	2 × 4 exercises (2 S + 2 P)	2 × 4 exercises (1 S + 3 P)	2 × 4 exercises (2 S + 2 P)
	W:R = 30:30	W:R = 30:30	W:R = 30:30
	total duration: 9 min	total duration: 9 min	total duration: 9 min
**WEEK 8**	2 × 4 exercises (2 S + 2 P)		2 × 4 exercises (4 S)
	W:R = 30:30		W:R = 30:30
	total duration: 9 min		total duration: 9 min
**WEEK 9**	2 × 4 exercises (4 S)	2 × 4 exercises (1 S + 3 P)	2 × 4 exercises (4 S)
	W:R = 30:30	W:R = 30:30	W:R = 30:30
	total duration: 9 min	total duration: 9 min	total duration: 9 min
**WEEK 10**	2 × 4 exercises (4 S)		2 × 4 exercises (4 P)
	W:R = 30:30		W:R = 30:30
	total duration: 9 min		total duration: 9 min

S: strength exercises, P: plyometric exercises, W:R: work-to-rest ratio.

**Table 3:** Changes in the examined parameters following 10 weeks of intervention in the training group (TG, *n* = 12) and the control group (CG, *n* = 11).

Measured Parameter	Group	Pre-Training	Post-Training	*p* (Interaction)	Cohen’s *d* (Pre vs. Post)	Δ Values (Pre vs. Post)	Cohens’ *d* of Δ Values Between Groups
**10 m Sprint (s)**	TG	2.05 ± 0.10	2.09 ± 0.11	0.709	0.44	0.04 ± 0.13	0.17
	CG	2.14 ± 0.07	2.17 ± 0.09		0.35	0.03 ± 0.08	
**Round-Off (s)**	TG	23.17 ± 2.56	20.97 ± 0.91	0.004	1.20	2.21 ± 2.00	1.47
	CG	23.09 ± 1.97	23.81 ± 1.86		0.40	0.72 ± 2.18	
**CMJ (cm)**	TG	24.00 ± 3.34	26.03 ± 4.68		0.52	2.0 ± 2.27	1.47
	CG	22.95 ± 3.66	21.82 ± 3.66	0.004	0.32	1.12 ± 2.22	
**R + L CMJ (cm)**	TG	24.99 ± 3.64	27.81 ± 4.40		0.73	2.82 ± 2.49	1.54
	CG	22.79 ± 3.31	22.20 ± 3.11	0.002	0.20	0.6 ± 2.10	
**DJ (cm)**	TG	22.12 ± 2.55	25.33 ± 4.15		0.98	3.21 ± 2.77	1.01
	CG	19.93 ± 3.98	20.30 ± 2.00	0.038	0.12	0.37 ± 3.10	
**Single-leg Jumping Agility (s)**	TG	17.91 ± 1.48	15.43 ± 0.86		2.15	2.48 ± 1.03	1.70
	CG	18.62 ± 1.30	18.09 ± 1.38	0.001	0.42	0.53 ± 1.36	

*Note*: CMJ: counter movement jump; R + L CMJ: sum of the right- and the left-leg counter movement jumps; DJ: drop jump.

## Supplementary Materials

The following are available online at https://www.mdpi.com/2075-4663/8/8/104/s1, Table S1: Plyometric and strength exercises used by the athletes of the training group.
