# Supplementary material for: Effects of Supplementary Strength–Power Training on Neuromuscular Performance in Young Female Athletes
Source: Sports (Basel). 2020 Jul 24;8(8):104. doi: 10.3390/sports8080104 (PMC7466675; doi:10.3390/sports8080104)
Supplement: Supplementary file 1 [file sports-08-00104-s001.pdf]

sports

MDPI

Table S1. Plyometric and strength exercises used by the athletes of the training group.

PLYOMETRIC EXERCISES	STRENGTH EXERCISES
Jumping lunges with resistance band	Pushups
Medicine ball (3 kg) throwing and catching while skipping	Lateral plank walks
Squat jumps with half-turn	Leg scissors with resistance band
Single leg jumping hip thrust	Half burpees with resistance band
Hands rebounds on plank position	Squat side-kicks with a 5 kg kettlebell
Snap down (gymnastics drill)	Hip thrust with resistance band
Squat jumps with resistance band	Overhead triceps extension with resistance band
Resistance band abductor squat jumps	Shoulder extension with resistance band from plank position
Clapping push-ups with feet elevated	Squat with a 5 kg kettlebell
Drop jumps from 45 cm	Lateral plank walking with push up
Burpees	Squat side-kicks with resistance band
Squat jumps over a step holding a 5 kg kettlebell	Rowing movement with resistance band
Step-up and jump with knee lift with resistance band	Straddle steps with a 5 kg kettlebell
Fast skipping with resistance band	Swings with a 5 kg kettlebell and resistance band
Reverse lunge, step-up and jump with knee lift	Elbow extensions with resistance band
Single leg side jumping with resistance band	Back scale with kettlebell 4/5kg
Box jumps with resistance band	Push-Press with resistance band
Fast skipping with resistance band	Single arm row with a 5 kg kettlebell
Single-leg box jumps to land on two-legs with resistance band	Plank with side knee flexion
Bulgarian split squat jump	Bulgarian split squat holding two 4 kg kettlebells
Tuck jumps over 6 low hurdles	Pistol squats (changing leg for each repetition)
Pistol squat and jump	Arm row holding two 4 kg kettlebells
Overhead press from squat position and jump holding two 4 kg kettlebells	Overhead press from squat position with two 4 kg kettlebells
